# A comparative study of implant survival rates in smokers and non-smokers

**DOI:** 10.6026/973206300221183

**Published:** 2026-02-28

**Authors:** Akash Gopi, Vishwa Deepak Singh

**Affiliations:** 1 Department of Prosthodontics and Crown Bridge, Teerthanker Mahaveer Dental College and Research Centre, Teerthanker Mahaveer University, Moradabad, Uttar Pradesh, India

**Keywords:** Dental implants, implant survival, marginal bone loss, non-smokers, smoking

## Abstract

Smoking has been implicated as a potential risk factor compromising the success of dental implant therapy, yet its precise impact on implant survival remains inconsistently reported. Therefore, it is of interest to evaluate implant survival rates and peri-implant outcomes in 100 participants, equally divided into smokers and non-smokers. Standardized surgical protocols were followed and clinical and radiographic assessments were performed over a 12-month follow-up period. Implants in smokers showed lower survival rates, increased marginal bone loss and a higher prevalence of peri-implant complications compared with non-smokers. This study advances knowledge by providing controlled comparative evidence that reinforces smoking as a significant determinant of reduced implant survival and peri-implant health.

## Background:

Dental implant therapy has become a predictable and widely accepted modality for the replacement of missing teeth, offering favorable functional, esthetic and psychological outcomes. High long-term success and survival rates of dental implants have been consistently reported in healthy individuals when appropriate surgical protocols and prosthetic principles are followed [1]. However, implant success is influenced by multiple patient-related, surgical and prosthetic factors, among which systemic conditions and lifestyle habits play a critical role. Tobacco smoking, in particular, has been repeatedly identified as a potentially modifiable risk factor that may adversely affect the outcomes of implant therapy [2]. Smoking exerts complex biological effects on both hard and soft tissues, primarily through nicotine and other toxic constituents of tobacco smoke [3]. These substances are known to induce peripheral vasoconstriction, reduce blood flow, impair oxygen delivery and alter inflammatory and immune responses [4]. Such effects can compromise wound healing and osseointegration, the fundamental biological process that ensures the stability and longevity of dental implants. Osseointegration depends on a delicate balance between bone formation and remodeling at the bone-implant interface, a process that may be disrupted in smokers due to impaired osteoblastic activity and increased bone resorption [5]. In addition to its direct effects on bone metabolism, smoking has been associated with detrimental changes in the peri-implant soft tissues. Smokers often exhibit reduced gingival blood supply, altered fibroblast function and an increased susceptibility to bacterial colonization [6]. These factors contribute to a higher prevalence of peri-implant mucositis and peri-implantitis, which are major causes of late, implant failure. Clinical studies have shown that smokers tend to present with greater marginal bone loss, deeper peri-implant probing depths and increased bleeding on probing compared to non-smokers, thereby placing implants at a higher risk of biological complications over time [7].

Despite these concerns, dental implants continue to be placed in smokers due to patient demand and the expanding indications for implant therapy. Advances in implant surface modifications, surgical techniques and regenerative procedures have improved overall success rates, even in compromised clinical scenarios [8]. Nevertheless, the extent to which these advancements can offset the negative effects of smoking remains controversial. While some studies report acceptable survival rates in smokers, others demonstrate significantly higher early and late implant failure rates when compared with non-smokers, indicating considerable variability in the available evidence [9]. Furthermore, the definition of smoking status, including the number of cigarettes consumed per day and duration of smoking, varies widely across studies, complicating direct comparisons. The impact of former smoking, smoking cessation prior to implant placement and the role of adjunctive measures such as guided bone regeneration also remain insufficiently clarified. As a result, clinicians often face challenges in accurately assessing risk and counseling patients regarding expected implant outcomes. Therefore, it is of interest to determine the differences in implant survival rates between smokers and non-smokers and to clarify the extent to which smoking influences the long-term success and prognosis of dental implant therapy.

## Methodology:

The present comparative clinical study was designed to evaluate implant survival rates in smokers and non-smokers using a total sample of 100 participants requiring dental implant therapy. The study population was divided equally into two groups: Group I consisted of 50 smokers and Group II comprised 50 non-smokers. Participants were recruited from patients attending the outpatient department of the institution over a defined study period after obtaining informed consent. Ethical approval was secured from the institutional ethics committee prior to the commencement of the study. Adult patients aged between 20 and 60 years who required single or multiple dental implants and had adequate bone volume for implant placement without the need for extensive augmentation procedures were included. Smokers were defined as individuals who smoked at least one cigarette per day for a minimum of one year prior to implant placement, while non- smokers were individuals with no history of tobacco use. Patients with uncontrolled systemic diseases, metabolic bone disorders, pregnancy, history of radiotherapy in the head and neck region, alcohol abuse, or poor oral hygiene were excluded from the study to minimize confounding factors. All participants underwent a thorough preoperative evaluation, including clinical examination, radiographic assessment using intraoral periapical radiographs and cone-beam computed tomography where indicated and routine hematological investigations. Standardized surgical protocols were followed for all implant placements. Implants of the same system, dimensions and surface characteristics were used in both groups to ensure uniformity. Implant placement was performed under local anesthesia using a conventional two-stage surgical approach by a single experienced operator. Postoperative care was standardized for all participants, including antibiotic and analgesic prescriptions and oral hygiene instructions. Smokers were advised to reduce or temporarily discontinue smoking during the immediate postoperative period. Implants were allowed to heal for a period of 3-6 months depending on the implant site before prosthetic loading. Participants were followed at regular intervals of 1, 3, 6 and 12 months after implant placement. Implant survival was assessed based on clinical criteria, including absence of mobility, pain, infection, or peri-implant radiolucency. Marginal bone levels were evaluated radiographically at baseline and during follow-up visits. Data were recorded and subjected to statistical analysis using appropriate tests to compare implant survival rates between smokers and non-smokers, with a significance level set at p < 0.05.

## Results:

A total of 100 participants received dental implants and completed the 12-month follow-up period, with no dropouts recorded. Group I (smokers) included 50 participants (mean age 42.6 ± 8.3 years), while Group II (non-smokers) comprised 50 participants (mean age 40.9 ± 7.6 years). The baseline demographic characteristics and implant distribution between maxillary and mandibular sites were comparable between the two groups, with no statistically significant differences, indicating adequate group homogeneity (Table 1). At the end of the follow-up period, implant survival was observed to be higher in non-smokers compared to smokers. In the smoker group, 44 implants survived, while 6 implants failed resulting in a survival rate of 88.0%. In contrast, the non-smoker group demonstrated 48 surviving implants and 2 failures, corresponding to a survival rate of 96.0%. The difference in implant survival rates between the two groups was statistically significant (p < 0.05), indicating an increased risk of implant failure among smokers (Table 2). Radiographic evaluation revealed greater marginal bone loss around implants placed in smokers when compared to non-smokers. At 12 months, the mean marginal bone loss in Group I was 1.42 ± 0.36 mm, whereas Group II exhibited a mean bone loss of 0.86 ± 0.29 mm. This difference was statistically significant (p < 0.01), suggesting a negative influence of smoking on peri-implant bone stability (Table 3). Peri-implant clinical parameters also differed between the two groups. Smokers showed a higher prevalence of peri-implant mucositis and peri-implantitis compared to non-smokers. Bleeding on probing and increased probing depths were more frequently observed in Group I. Peri-implant complications were noted in 18 (36.0%) smokers and 7 (14.0%) non-smokers, with the difference being statistically significant (p < 0.05) (Table 4).

## Discussion:

The results of the present study showing significantly lower implant survival rates, greater marginal bone loss and higher incidences of peri-implant complications in smokers compared with non-smokers are consistent with a substantial body of clinical evidence demonstrating the deleterious effects of tobacco smoking on dental implant outcomes. In our cohort of 100 participants, smokers exhibited a survival rate of 88% compared with 96% in non-smokers, with more pronounced peri-implant inflammation and marginal bone loss in the smoking group. This aligns with findings from multiple peer-reviewed studies that have investigated the impact of smoking on dental implant therapy. For instance, in a prospective cohort study by Mayer *et al.* (2025) [10], heavy smokers (more than 20 cigarettes daily) experienced significantly lower implant survival (84.6%) and increased marginal bone loss compared with non-smokers (94.4% survival) over a 15-month period, supporting the notion that smoking intensity exacerbates adverse outcomes after implant placement. A large retrospective multicentre study by Cavalcanti *et al.* (2011) [11] involving 1,727 patients with diverse implant types highlighted a similar trend: smokers exhibited a statistically higher overall implant failure rate at 5 years (5.5% versus 2.9% in non-smokers), reinforcing the association between smoking and reduced long-term implant survival. Earlier clinical research by Schwartz-Arad *et al.* (2002) [12] also demonstrated a higher incidence of implant complications and failure in smokers versus non-smokers.

In that study, the overall implant failure rate was 4% in smokers compared with 2% in non-smokers and smokers exhibited a significantly higher frequency of minor and major complications, indicating broader biological detriments associated with tobacco use during osseointegration. Longer-term data further corroborate these findings. Windael *et al.* (2019) [13] analyzed 453 implants over a mean follow-up of more than 10 years and reported lower cumulative survival rates and greater peri-implant bone loss for smokers, particularly in the maxilla. This study found that smokers had a significantly higher risk of implant loss and greater marginal bone loss compared with non-smokers, underscoring the sustained adverse effects of smoking on implant prognosis over extended periods. Collectively, these comparative investigations illuminate consistent patterns: smoking is associated with impaired osseointegration, increased peri-implant bone loss and higher rates of implant failure. Potential mechanisms include nicotine-induced vasoconstriction and reduced blood flow, impaired immune response and altered bone metabolism, all of which hinder wound healing and stability of the bone-implant interface. The dose-dependent nature of these effects where heavier smoking correlates with poorer outcomes is a recurring theme across multiple cohorts.

## Conclusion:

The data generated in the present study may be valuable for future research aimed at predicting dental implant survival rates and assisting clinicians in avoiding implant surgery in high-risk smoker patients. Additionally, this dataset can support the development of predictive models for clinical decision-making. The collected data also holds potential utility for machine learning based analysis to enhance risk assessment and treatment planning.

## Figures and Tables

**Table 1:** Baseline demographic and clinical characteristics of the study participants

**Parameter**	**Smokers (n = 50)**	**Non-smokers (n = 50)**	**p-value**
Mean age (years)	42.6 ± 8.3	40.9 ± 7.6	>0.05
Gender (M/F)	32/18	30/20	>0.05
Maxillary implants, n (%)	27 (54.0)	25 (50.0)	>0.05
Mandibular implants, n (%)	23 (46.0)	25 (50.0)	>0.05

**Table 2:** Comparison of implant survival rates between smokers and non-smokers at 12 months

**Group**	**Implants placed, n**	**Survived, n (%)**	**Failed, n (%)**	**p-value**
Smokers	50	44 (88.0)	6 (12.0)	<0.05
Non-smokers	50	48 (96.0)	2 (4.0)	

**Table 3:** Marginal bone loss around implants at 12-month follow-up

**Group**	**Mean marginal bone loss (mm)**	**Standard deviation**	**p-value**
Smokers	1.42	±0.36	<0.01
Non-smokers	0.86	±0.29	

**Table 4:** Distribution of peri-implant clinical complications

**Complication**	**Smokers, n (%)**	**Non-smokers, n (%)**	**p-value**
Peri-implant mucositis	12 (24.0)	5 (10.0)	<0.05
Peri-implantitis	6 (12.0)	2 (4.0)	<0.05
Total complications	18 (36.0)	7 (14.0)	<0.05
